# Typical Wind Shear Simulation and Detection Analysis Based on Coherent Doppler Wind Lidar

**DOI:** 10.3390/s26051643

**Published:** 2026-03-05

**Authors:** Yuanyuan Wei, Jinlong Yuan, Chaoyong Chen, Tengfei Wu, Zikang Tong

**Affiliations:** 1 Changcheng Institute of Metrology & Measurement, Aviation Industry Corporation of China (AVIC), National Key Laboratory of Metrology and Calibration, Beijing 100095, China; weiyy@radi.ac.cn (Y.W.); chency035@avic.com (C.C.); wutf@avic.com (T.W.); tongzk@avic.com (Z.T.); 2 School of Atmospheric Physics, Nanjing University of Information Science & Technology (NUIST), Nanjing 210044, China

**Keywords:** wind lidar, microburst, wind shear, plateau airport, aviation safety, CFD

## Abstract

**Highlights:**

This study analyzed wind shear identification from coherent Doppler wind lidar (CDWL) detection results under typical airflow disturbances. Numerical simulation showed that the double-slope algorithm is superior for peak-characteristic wind fields, while the least squares algorithm fits stable-linear-gradient wind fields. Field experiments were carried out at Panzhihua Baoanying Plateau Airport using a self-developed long-range CDWL. After beam calibration and anemometer data verification, the “least squares + double-slope” combined scheme was applied to the typical wind shear cases, and its effectiveness and reliability were confirmed by aircraft crew reports.

**What are the main findings?**

- Different wind shear algorithms are suitable for different flow field characteristics, based on numerical simulation.
- A self-developed long-range wind lidar can successfully identify wind shear at an airport.

**What are the implications of the main findings?**

- The “least squares + double-slope” identification scheme provides a generalizable technical framework for multi-type wind shear detection. This implies that future lidar-based wind shear detection systems can adopt modular algorithm combinations to adapt to diverse atmospheric scenarios.
- The airport verification results indicate that CDWL has high sensitivity to sudden wind speed changes and can capture wind shear information, which supports the deployment of CDWL as a reliable wind shear monitoring tool at complex airports.

**Abstract:**

To enhance the accuracy of wind shear identification by coherent Doppler wind lidar (CDWL), it is necessary to clarify the variation characteristics of CDWL detection results under typical airflow disturbance conditions. This study first numerically simulated typical wind shear fields and generated the Plane Position Indication (PPI) results of CDWL through coordinate projection. Then, it compared the performance of the double-slope algorithm and the least squares algorithm on wind shear identification from the PPI data. The results showed that for wind fields with significant peak characteristics, the double-slope algorithm can more sensitively identify wind shear near the peak, compared with the least square algorithm. In contrast, for wind fields with stable, continuous and linear gradient characteristics, the least squares algorithm can better suppress noise and fit the wind speed gradient changes. Finally, a self-developed long-range CDWL was used to conduct wind shear detection experiments at a plateau airport. After the CDWL beam position was calibrated, its data were compared with those from the anemometer. The “least square + double-slope” scheme was adopted to analyze the typical wind shear case, and the effectiveness and reliability of the identification scheme were verified in combination with an aircraft crew report.

## 1. Introduction

In meteorology, wind shear refers to the difference in the average wind vector between two points in space [1]. Low-altitude wind shear usually occurs below 600 m in altitude and is characterized by short duration, small scale and high intensity [2,3,4]. It poses a serious threat especially during the landing and takeoff of aircraft [5,6,7]. Therefore, the detection and identification of low-altitude wind shear are of great significance for ensuring aviation safety. Meanwhile, identifying the characteristics of wind shear is also helpful for studying the process of weather changes [8].

There are various means for wind shear detection. An anemometer is usually placed on airport runways and can only monitor wind shear on the surface at a single point. The wind shear along the flight path can be calculated in real time by using the data from conventional aircraft sensors, such as airspeed tubes, vertical gyroscopes, and stall warning airflow sensors. However, such systems only issue warnings after the aircraft enters wind shear. It can issue a warning within the crucial few seconds when the pilot is not aware of wind shear, but it cannot predict the wind shear in advance [1,9,10]. Ground-based and airborne Doppler weather radar can detect wind shear by processing the Doppler signals of precipitation particles and cloud droplets. By using antennas and transmitters operating in specific bands, a downburst detection and warning algorithm was designed. The airborne system scans a certain range of airspace in front of the aircraft. When wind shear is detected, the relevant area is displayed on the navigation display unit, accompanied by audio warnings and visual prompts in the cockpit. This system can provide pilots with advance reaction time, but it relies on radar detection of water droplets and ice particles, which fails under dry and clear sky conditions [11,12,13].

Coherent Doppler wind lidar (CDWL) is widely regarded as a more effective device for observing wind shear in clear skies. CDWL is based on the principle of Doppler frequency shifts of laser pulses. It continuously measures wind speed information with spatiotemporal resolution according to the laser backscattering echoes of aerosols in the air. Compared with traditional methods, CDWL can provide continuous coverage of the wind field variation characteristics at the runway side of the airport, with a flexible scanning mode. Thus, it can provide important observational data support for early warning of hazardous wind fields at airports, such as excessive tailwind, wind shear, and clear sky turbulence. Various wind shear identification algorithms have been developed based on CDWL [7,14,15,16,17,18,19,20,21]. Among them, the single- and double-slope algorithm uses the single- and double-slope characteristics of wind speed variation in wind profiles to determine wind shear. However, this algorithm has high requirements for the shape of wind profiles. If the manifestation of wind shear is not the typical single- or double-slope mode, there may be cases of misjudgment. The least squares algorithm uses the linear fitting feature and radial distribution of lidar data to calculate the one-dimensional radial and azimuth shears separately, then fuses them to derive the two-dimensional synthetic wind shear. This algorithm has a more stable recognition effect on wind shear with continuous linear gradient features, but it has limitations in recognizing sudden wind shear.

Clarifying the spatial distribution characteristics of various typical forms of wind shear by simulation can further improve the accuracy and reliability of wind shear identification. Considering that different wind shear algorithms are suitable for different flow field characteristics, an identification scheme providing a generalizable technical framework for multi-type wind shear detection is proposed. This paper combines typical wind shear simulation data to conduct tests on this wind shear detection scheme. Then, self-developed long-range CDWL is used to conduct wind shear detection experiments at a plateau airport, Panzhihua Baoanying Airport in Sichuan Province.

## 2. Simulation and Analysis of Typical Wind Shear

### 2.1. Microburst Simulation

A microburst is a strong downward air current that spreads in all directions after reaching the ground, forming a unique wind field structure, and is a common weather phenomenon in areas prone to thunderstorms. Microbursts consist of downdraft and outward-diffusing horizontal airflow. Compared with simplified microburst numerical models such as the Oseguera & Bowles (OB) model [22,23,24], computational fluid dynamics (CFD) enables realistic simulation of the unsteady, highly sheared and turbulent flow structures of microbursts by solving the Navier–Stokes equations with appropriate turbulence closures. Thus, CFD simulation was performed with the embedded Large Eddy Simulation (LES) turbulence model to provide high-fidelity microburst wind fields for wind lidar verification. A cuboid computational domain was defined as 3000 m (length) × 3000 m (width) × 900 m (height). A velocity inlet with a radius of 300 m and downward velocity of 25 m/s was deployed in the upper boundary to replicate the core descending airflow of the microburst. The ground boundary was set as a no-slip wall, while the far-field boundaries adopted pressure far-field conditions.

**Figure 1:**
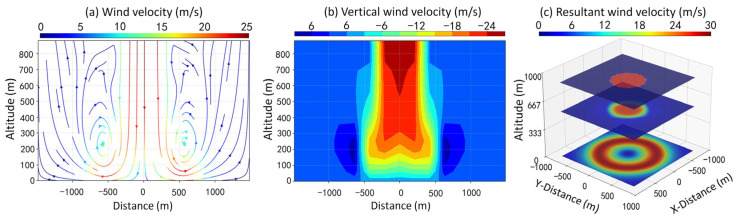
The CFD model simulation of (**a**) the vector wind velocity and (**b**) vertical velocity distribution of a microburst in the *x*-*z* plane. (**c**) The resultant velocity of the downdraft in the 3D coordinate system.

The three-dimensional wind field of the microburst was simulated as shown in Figure 1. Figure 1a and Figure 1b represent vertical slices of the vector wind velocity and vertical velocity, respectively. The distance–altitude coordinate axis corresponds to the *x*-*z* plane in three-dimensional space. The characteristic of the sinking diffusion is illustrated by the vector velocity distribution in Figure 1a. A prominent downdraft is centered at *x* ≈ 0, with air converging from high altitude toward the ground, then spreading horizontally to the sides. Distinct vortical structures appear on both sides, with spiraling flow patterns characteristic of microburst dynamics. Meanwhile, in Figure 1b, a strong downdraft (red region, with vertical wind velocity of about −24 m/s; negative sign denotes downward motion) dominates the central column, extending from high altitude down to about 200 m above the ground. Updraft regions (blue, with vertical wind velocity of about 6 m/s) appear on both sides near the ground (*x* ≈ ±500 m, *z* ≈ 200 m~300 m), forming complementary circulation with the central downdraft. Horizontal slices of the resultant velocity in the 3D coordinate system are displayed in Figure 1c, which presents the conical/drain-shaped spatial structure of the microburst.

### 2.2. Wind Shear Identification and Analysis

The wind shear intensity is calculated based on the ratio of the wind vector difference to the distance between the two points. The center (*x_ko_*, *y_ko_*) is set as the origin *O_k_*, the direction parallel to the reference (such as runway) is set as the *X_k_* axis, and the direction perpendicular to the reference is set as the *Y_k_* axis. Then, the heading wind, *U_k_*, and crossing wind, *V_k_*, in the reference coordinates *X_k_O_k_Y_k_* are expressed as [25]

(1) $$U_{k}=u\cos\theta_{k}+v\sin\theta_{k}$$

(2) $$V_{k}=-u\sin\theta_{k}+v\cos\theta_{k}$$

where $\theta_{k}$ is the angle between the reference coordinate and the lidar coordinate. Then, the headwind change of any two points A and B (30 m apart in this study) in the monitoring along the glide path is calculated as

(3) $$U_{a,b}=U_{a}-U_{b}$$

The wind shear intensity grade standards are given by the International Civil Aviation Organization [1] (as shown in Table 1), in which wind shear is classified into five different grades based on intensity.

The double-slope algorithm [20] utilizes the slope peak characteristics of wind profiles to determine wind shear based on the variation in wind speed. The least squares algorithm [26] takes advantage of the linear fitting feature and combines the radial distribution characteristic of lidar data to obtain the two-dimensional synthetic wind shear.

The main difference between the two lies in their objective functions. The objective function of the least squares algorithm is composed of a quadratic function:

(4) $$min\;J=\overset{N}{\underset{j=1}{\sum }}{\left[w_{j}-\left(a+b\cdot l_{j}+cl_{j}^{2}\right)\right]}^{2}$$

where *w_j_* represents the wind speed value at the *j*th sampling point, and *l_j_* represents the distance from the *j*th sampling point to the core point of the wind shear. *a*, *b*, and *c* are the fitting parameters. The wind shear intensity is obtained from the first derivative of the quadratic function.

Meanwhile, the objective function of the double-slope algorithm is made up of two linear functions. In the following function, *l*_0_ represents the point of sudden change in the wind speed.

(5) $$min\;J=\underset{l_{j}\leq l_{0}}{\sum }{\left[w_{j}-\left(a_{1}+b_{1}l_{j}\right)\right]}^{2}+\underset{l_{j}>l_{0}}{\sum }{\left[w_{j}-\left(a_{2}+b_{2}l_{j}\right)\right]}^{2}$$

where the fitting coefficients ($a_{1},b_{1},a_{2},b_{2}$) can be solved by solving the multi-objective function. Then, the wind shear intensity of the wind shear can be derived from $b_{1}$ and $b_{2}$.

The double-slope algorithm and the least squares algorithm were both adopted to identify wind shear and calculate the wind shear intensity in the microburst wind field simulated in Section 2.1. The results of the Plane Position Indication (PPI) scan data projected at different elevation angles are shown in Table 2 below. Compared with those of the least squares algorithm, the fitting residuals of the double-slope algorithm were significantly smaller, with better performance. Additionally, wind shear intensity increased with decreasing PPI scan elevation angle. Therefore, for a microburst near the airport runway and its extension, the lower the flight trajectory, the greater the wind shear intensity encountered.

**Figure 2:**
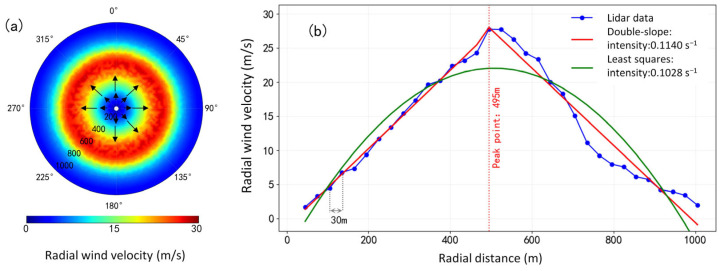
(**a**) Lidar PPI scanning radial wind speed at a 3° elevation angle. (**b**) The results of wind shear identification from radial data based on the double-slope algorithm and the least squares algorithm.

The PPI scanning mode sets the lidar to a fixed elevation angle and performs a conical surface scan in a 360° orientation. To project the simulated three-dimensional wind field data onto the PPI scanning plane, lidar was deployed at the center of the simulated wind field. The radial wind from PPI scanning with a 3° elevation is shown in Figure 2a. Figure 2b shows fitting curves from the two algorithms. The blue dots represent the radial wind speed at each distance gate, with the maximum wind speed occurring at 495 m. The red curve and the green curve are the fitting results of the double-slope algorithm and the least squares algorithm, respectively. For wind fields with significant peak characteristics, the double-slope algorithm can achieve linear fitting of the data on both sides of the peak, thereby more sensitively identifying the wind shear near the peak. Conversely, the quadratic curve fitted by the least squares algorithm overly smoothens the rapid changes in wind speed, resulting in an underestimation of the wind shear intensity.

**Figure 3:**
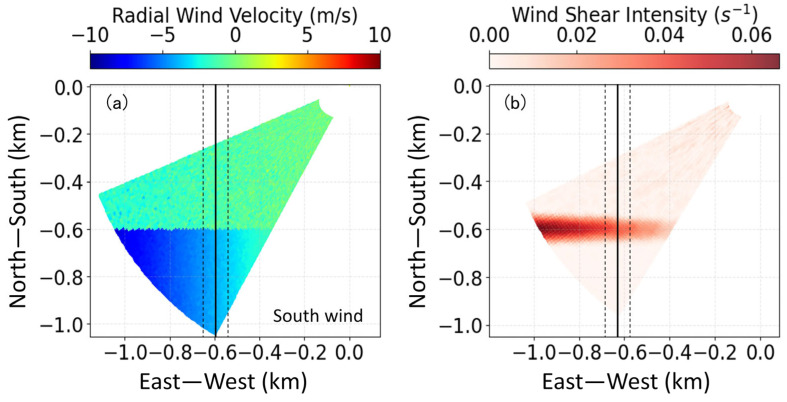
(**a**) Numerical simulation of radial wind speed in glide path scanning. (**b**) Corresponding wind shear intensity using least squares algorithm. The solid line in the middle (x = −0.6 km) is the extension center line of the runway, and the other two dotted lines are located 50 m either side of the extension center line to take into account the uncertainty of the aircraft’s flight path.

**Figure 4:**
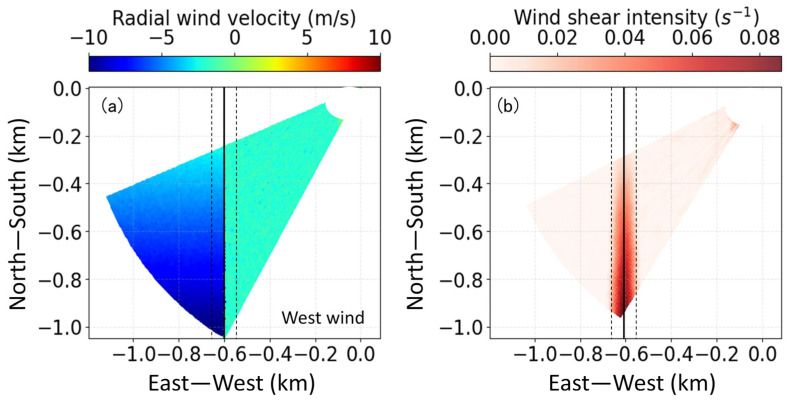
(**a**) Numerical simulation of radial wind speed in glide path scan. (**b**) Wind shear identification crossing runway direction. The solid line in the middle (x = −0.6 km) is the extension center line of the runway, and the other two dotted lines are located 50 m either side of the extension center line to take into account the uncertainty of the aircraft’s flight path.

In addition to the aforementioned microburst with peak structure characteristics, there also exist continuous linear and gradually changing wind shear, such as tailwind shear, headwind shear, and crosswind shear, relative to aircraft motion. The identification results for wind shear along the runway (tailwind shear or headwind shear) and perpendicular to the runway (crosswind shear) based on simultaneous simulation are shown in Figure 3 and Figure 4. The solid line in the middle (*x* = −0.6 km) is the extension center line of the runway, and the other two dotted lines are located 50 m either side of the extension center line to take into account the uncertainty of the aircraft’s flight path.

In Figure 3a, the negative radial wind speed indicates that the airflow is moving towards the lidar, with the lidar position set at the origin (0, 0). Suppose the landing direction of the aircraft is along the *Y*-axis. For an aircraft landing from north to south, there is significant headwind shear along the runway direction. Figure 3b shows the corresponding headwind shear intensity results using the least squares algorithm. The shear line *y* = −0.6 km is accurately identified, and the shear intensity values are consistent with the simulated wind speed differences.

The typical crosswind field is simulated in Figure 4a, with wind speed variations in the east–west direction (*x*-axis). When an aircraft flies along the positive *y*-axis (from south to north), the crosswind direction decreases from the west (left side wind). The crosswind shear line is *x* = −0.6 km. The corresponding wind shear intensity results using the least squares algorithm are well located at the crosswind shear line, as shown in Figure 4b. Crosswind from south to north weakens on the left side of the runway, with the shear intensity decreasing accordingly, verifying the effectiveness of crosswind shear recognition. The least squares algorithm has good performance in the identification of tailwind shear, headwind shear and crosswind shear, which is related to the linear stability of the wind characteristics of the wind field. It can suppress noise well and fit the changes in the wind speed gradient.

The double-slope algorithm and the least squares algorithm are suitable for different scenarios. Therefore, it is necessary to combine the two algorithms to avoid the omission of wind shear detection. In detail, the wind shear identification scheme based on CDWL can be divided into two steps. Firstly, to preliminarily detect continuous-gradient wind shear, the least squares detection algorithm is adopted to identify tailwind/headwind/crosswind wind shear. Then, the double-slope algorithm is adopted to identify peak-structure wind shear (such as microbursts). In this way, the location and intensity of wind shear can be scientifically identified, reducing missed detections and underestimations.

## 3. Detection and Verification of Wind Shear by CDWL at Plateau Airports

### 3.1. Field Test Site and Instrument

**Figure 5:**
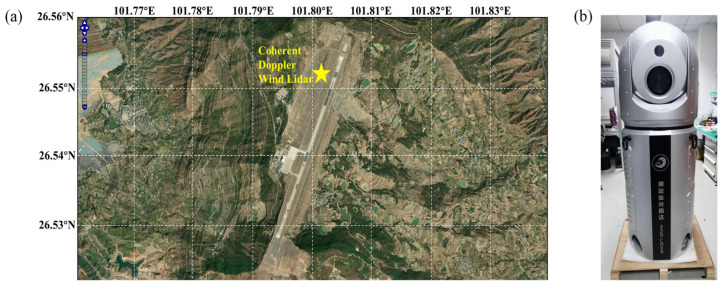
(**a**) An image of the geographical location of Panzhihua Baoanying Airport; (**b**) the CDWL.

Panzhihua Baoanying Airport is located on the east side of Baoanying Mountain Peak in Jinjiang Town, on the south bank of the Jinsha River, Panzhihua City, Sichuan Province, China (101.80° E, 26.54° N), as shown in Figure 5a. This airport is a 4C-level domestic regional airport, with an altitude of 1980.2 m in a plateau area. Due to its unique terrain and meteorological conditions, the wind field exhibits distinct regional characteristics, making it one of the most challenging airports for flight operations in China. The runway is 2800 m long and 45 m wide. In this field test, the CDWL (Figure 5b) was deployed 100 m from the runway. The scanning mode was set to PPI, the scanning elevation angle was 3°, and the interval of the azimuth angle was 3°. The detailed parameters of the lidar are shown in Table 3.

### 3.2. Calibration of the CDWL

The positional accuracy of wind shear detection depends on the range resolution and angular resolution of the lidar and is also related to the distance and angular detection accuracy. The distance and angle measurements of lidar are not completely independent, and there is measurement error coupling between them. For instance, angular pointing deviation causes the actual irradiation position of the laser beam to shift, thereby introducing indirect errors into distance measurement. Conversely, systematic deviations in distance measurement may also affect the accuracy of angle calculation. In this study, “range–angle“ joint calibration was carried out for the CDWL. The essence is to establish an error model and acquire high-precision target reference data via a higher-precision laser tracker. This enables the inversion and correction of systematic, random and coupling errors in distance and angle and ultimately calibrates the mapping between measured and true physical values. The true distance, elevation, and azimuth angle (*R_t_*, *θ_t_*, *ϕ_t_*) of the target were obtained by a high-precision laser tracker. The measured distance and angle (*R_m_*, *θ_m_*, *ϕ_m_*) of the target were obtained by lidar. By introducing error correction terms, the actual measurement model was established as shown in the following Formula (6):

(6) $$\left\{\begin{array}{c} R_{m}=R_{t}+△R\left(\theta ,\phi \right)+\delta R\\ \theta_{m}=\theta_{t}+△\theta \left(\phi \right)+\delta \theta \\ \phi_{m}=\phi_{t}+△\phi \left(\theta \right)+\delta \phi \end{array}\right.$$

here, Δ*R*(*θ*, *ϕ*) represents the distance coupling error related to the angle, such as the slant deviation caused by angle offset. Δ*θ*(*ϕ*) and Δ*ϕ*(*θ*) represent the systematic deviations in the angle itself, related to installation eccentricity and shafting. *δR*, *δθ*, and *δϕ* represent random errors, which are usually suppressed by averaging multiple measurements.

The deviation between the true coordinates and the calculated measured coordinates was fitted to an error curve using the least square algorithm, and the error correction term function was subsequently derived via inversion. The linear coupling model was adopted to fit the coupling error of distance varying with angle, and the expression is as follows:

(7) $$\left\{\begin{array}{c} △R=dR_{0}+dR_{1}\cdot \theta +dR_{2}\cdot \phi \\ △\theta =d\theta_{0}+d\theta_{1}\cdot \phi \\ △\phi =d\phi_{0}+d\phi_{1}\cdot \theta \end{array}\right.$$

here, the distance deviation Δ*R*(*θ*, *ϕ*) and angle deviations Δ*θ*(*ϕ*) and Δ*ϕ*(*θ*) are the systematic deviations of the lidar. *dR*_0_ represents the distance offset coefficient. *dR*_1_ and *dR*_2_ represent the distance–elevation angle and distance–azimuth angle coupling coefficients. *dθ*_0_ represents the elevation angle offset coefficient. *dθ*_1_ represents the elevation–azimuth angle coupling coefficient. *dϕ*_0_ represents the azimuth offset coefficient. *dϕ*_1_ represents the azimuth–elevation angle coupling coefficient.

**Figure 6:**
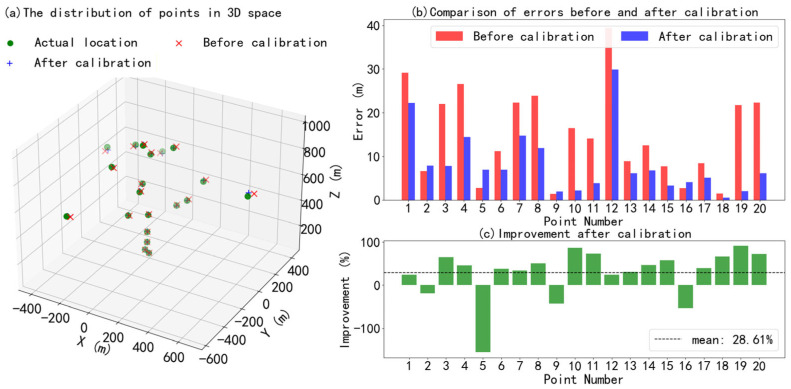
(**a**) Comparison of point distribution in 3D space (actual location, before calibration, after calibration). (**b**) Error comparison for each calibration point. (**c**) Error improvement percentage for each calibration point.

To demonstrate the calibration effect of the “range–angle“ joint calibration method, the actual positions and the lidar measurement position (including systematic errors and random noise before calibration) of 20 calibration points in three-dimensional space are shown in Figure 6. The simulated random errors of the position of the laser beam follow a normal distribution in this study. At the same time, the systematic deviation exhibits a two-dimensional cosine fluctuation with respect to the zenith angle and azimuth angle and has a fixed positive offset. Δ*θ*(*ϕ*) fluctuates sinusoidally with the azimuth angle, with a fixed positive offset. Δ*ϕ*(*θ*) fluctuates as a cosine function with respect to the elevation angle and has a fixed positive offset. The distance range from the calibration points to the lidar is 10 m to 1000 m, the elevation angle range is 0° to 90°, and the azimuth angle range is 0° to 360°.

Figure 6 shows that calibration can significantly reduce location measurement errors. The average error before calibration was 15.07 m, and the average error after calibration was 8.23 m, as shown in Figure 6b. The maximum error improved by 90.82%, and the average error improved by 28.61%, as shown in Figure 6c. The “range–angle” joint calibration method can correct the scanning trajectory deviation caused by various influencing factors, including center axis deviation of the lidar, system error of the servo drive, processing error of the optical lenses, deformation error caused by temperature drift, and so on.

### 3.3. Comparison of Wind Speed and Direction Data

**Figure 7:**
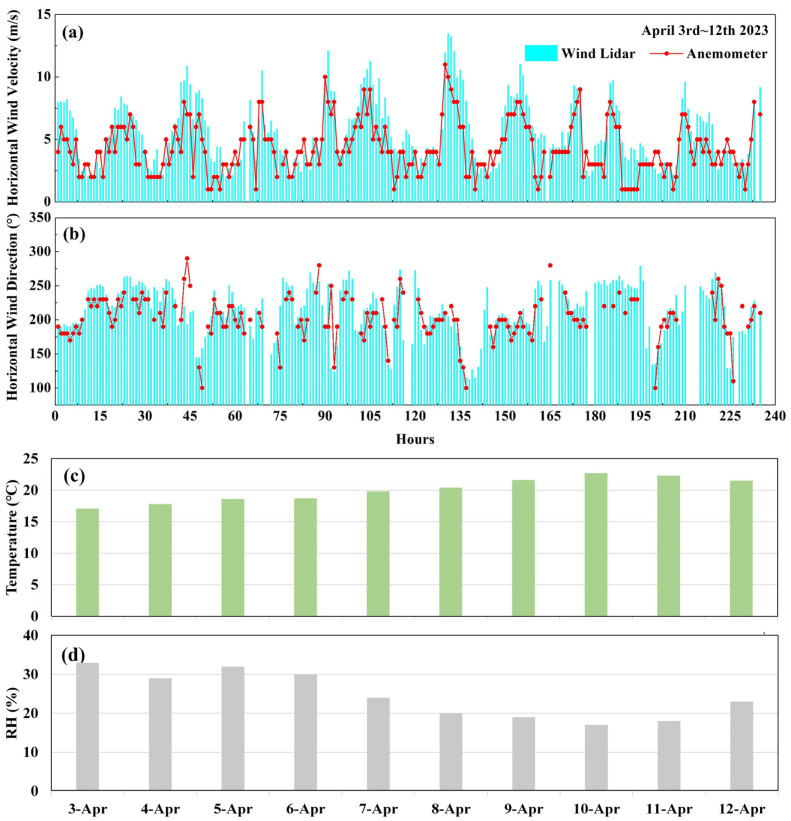
A comparison of trend consistency from the CDWL and anemometer for (**a**) wind speed and (**b**) wind direction. The daily mean (**c**) temperature and (**d**) RH data from the surface sensors.

The changing trends in the measured values from the airport anemometer and the CDWL are compared in Figure 7a,b. The cup-type anemometer at the airport was installed at a height of 10 m above the ground and 600 m away from the laser radar in the direction of the airport runway. The discrimination of the airport anemometer is 1 m/s with an interval of 10°, and the time resolution is 1 h. The two trends show good consistency and a significant positive correlation. The correlation coefficients R^2^ of wind speed and wind direction from the anemometer and CDWL are 0.79 and 0.70, respectively. The RMSE values of wind speed and wind direction are 1.64 m/s and 29.33°, respectively. Compared with the measurement results from the anemometer, the wind speed value from the CDWL is significantly higher. This is primarily because the 3° elevation angle of the CDWL makes its measurement height approximately 30 m higher than that of the anemometer.

Temperature and relative humidity (RH) are the key factors influencing the thermal structure and stability of the atmosphere. The formation, intensity and maintenance of wind shear directly depend on the coupling effect of the thermal and dynamic fields of the atmosphere. The daily mean temperature and RH are shown in Figure 7c,d. The average temperature and RH were 20.05 °C and 24.5% during the experiment period. The standard deviations were 1.95 °C and 5.98% respectively. The temperature condition was stable and the surface air was relatively dry. In general, humidity influences the performance of the lidar, while elevated temperatures boost atmospheric turbulence and thus lower the data consistency between the anemometer and the lidar.

**Figure 8:**
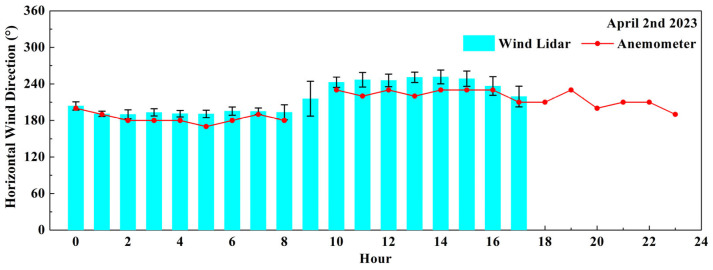
The hourly variation in wind direction data obtained by the wind lidar (blue column) and the anemometer (red dot line) on 2 April 2023.

The CDWL and anemometer can simultaneously detect significant changes in wind direction (VRB). According to the civil aviation meteorological ground observation specifications, the term VRB will be used when the following situations occur. During the observation period, if the wind direction change exceeds 180°, or the direction change is 60–180° with an average wind speed less than 2 m/s, it is defined as an unstable wind direction. Figure 8 shows the comparison results for wind direction data on 2 April 2023. The red dots and lines represent the hourly average wind direction from the anemometer, from 0:00 to 23:00. The blue bars represent the hourly average wind direction from the CDWL, from 0:00 to 17:00, and the black error bars represent the hourly standard deviation of the wind direction data, with an average of 9.94°. Due to instrument maintenance, no lidar data were collected after 17:00. The average wind direction of the anemometer during this period was 202.94°, and VRB was detected by the anemometer at 9 a.m. The average wind direction of the CDWL during this period was 215.93°; the hourly average standard deviation of the wind direction was the largest at 9 a.m., reaching 28.53°. This indicates that the CDWL and the wind anemometer simultaneously captured the phenomenon of an unstable wind direction.

### 3.4. Wind Shear Detection and Verification

**Figure 9:**
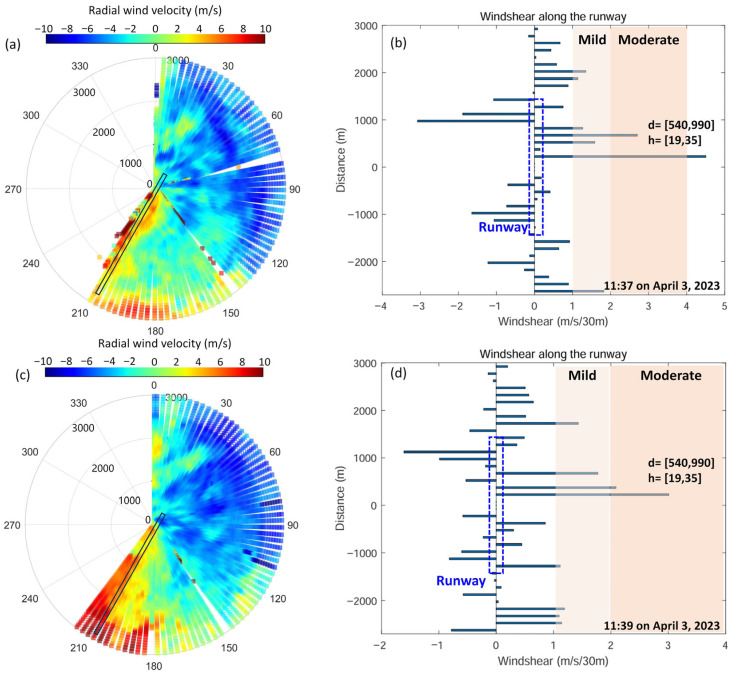
(**a**,**c**) The PPI scanning data and (**b**,**d**) the detected wind shear results at 11:37 and 11:39 on 3 April 2023. The black rectangle indicates the location of the airport runway.

During this field test, at 11:37 to 11:39 on 3 April 2023, the CDWL detected mild to moderate low-altitude wind shear and triggered an alarm with the “least squares + double-slope” combined method. Figure 9a and Figure 9c show the lidar PPI scanning results at 11:37 and 11:39, respectively, with the lidar located at the origin of the polar coordinate system. Figure 9b,d show the wind shear detection results along the runway direction, with the runway center located at the origin of the Cartesian coordinate system. The identified wind shear was located in the north of the airport, 540 m to 990 m away from the runway center, with a height range of 19 m to 35 m. Meanwhile, a flight crew report was recorded by the airport’s meteorological observatory: “At 11:37 to 39 on 3 April 2023, the flight encountered low-altitude wind shear 50 feet above the north runway at this airport, which lasted for 4 s to 5 s”. Thus, the wind shear alarm from the CDWL was verified by the flight crew.

## 4. Discussion and Conclusions

This study simulated the atmospheric wind field data of typical wind shear, including microburst, tailwind/headwind shear and crosswind shear. By comparing the wind shear detection algorithms, it was found that for wind fields with significant peak characteristics, the double-slope algorithm can sensitively identify the wind shear near the peak. The least squares algorithm can better suppress noise and fit a stable linear gradient change in wind speed. Thus, the “least squares + double-slope” combined method is recommended to screen multiple types of wind shear, as demonstrated in an experiment at Panzhihua Plateau Airport. With calibrated and verified CDWL, a wind shear case was well detected and identified and was consistent with an aircraft crew report. The effectiveness and reliability of the identification scheme were verified in combination with simulations and airport experiments. However, additional datasets of airport wind shear cases are needed to further optimize the method and strengthen its robustness. In practical applications, wind shear identification is subject to interference from turbulent components. Although such interference can be suppressed by smoothing methods, developing more robust turbulence filtering strategies warrants further investigation in future research. Moreover, enhancing the spatial–temporal resolutions of CDWL can refine the accuracy of the proposed wind shear identification approach, which in turn strengthens its capability in ensuring aviation safety.

## Figures and Tables

**Table 1:** List of wind shear intensity standards [1].

Windshear Level	Windshear (m × s^−1^ × 30 m^−1^)	Windshear Intensity (s^−1^)
Mild	<2.0	<0.067
Moderate	2.1~4.0	0.068~0.133
Strong	4.0~6.0	0.134~0.200
Severe	>6.0	>0.200

**Table 2:** The wind shear intensity calculated from multi-elevation PPI scanning data.

Elevation Angles (°)	Wind Shear Intensity (s^−1^)		Fitting Residuals (m × s^−1^)	
	Double-Slope Algorithm	Least Squares Algorithm	Double-Slope Algorithm	Least Squares Algorithm
0.5	0.1129	0.1032	2.53	10.81
3.0	0.1140	0.1028	2.76	11.35
5.0	0.1130	0.1032	3.02	11.56
10.0	0.1117	0.1002	3.03	11.33
20.0	0.1057	0.0945	1.56	7.57

**Table 3:** Detailed parameters of the coherent Doppler wind lidar.

Parameters	Values
Radial detection range	0~15 km
Radial velocity range	±50 m/s
Range resolution	30 m
Temporal resolution	1 s
Azimuth range	0~360°
Elevation range	0~180°
Scanning accuracy	±0.1°
Weight	120 kg
Power	250 W
Volume	0.4 m × 0.4 m × 1.6 m

## Data Availability

Data underlying the results presented in this paper can be obtained from the authors upon reasonable request.
